# The Relationship between Phytoplankton Evenness and Copepod Abundance in Lake Nansihu, China

**DOI:** 10.3390/ijerph13090855

**Published:** 2016-08-26

**Authors:** Wang Tian, Huayong Zhang, Lei Zhao, Xiang Xu, Hai Huang

**Affiliations:** Research Center for Engineering Ecology and Nonlinear Science, North China Electric Power University, Beijing 102206, China; tianwang870822@163.com (W.T.); zhaolei@ncepu.edu.cn (L.Z.); xuxiang229@163.com (X.X.); bjecology@gmail.com (H.H.)

**Keywords:** phytoplankton biomass, species richness, evenness, copepod abundance, ecosystem functioning

## Abstract

The relationship between biodiversity and ecosystem functioning is a central issue in ecology. Previous studies have shown that producer diversity can impact the consumer community via predator-prey interactions. However, direct observations of this relationship remain rare, in particular for aquatic ecosystems. In this research, the relationship between phytoplankton diversity (species richness and evenness) and the abundance of copepods was analyzed in Lake Nansihu, a meso-eutrophic lake in China. The results showed that copepods abundance was significantly decreased with increasing phytoplankton evenness throughout the year. However, both species richness and phytoplankton biomass showed no significant relationship with the abundance of copepods. Canonical correspondence analysis revealed that phytoplankton evenness was negatively correlated with *Thermocyclops kawamurai*, *Cyclops vicinus*, *Eucyclops serrulatus*, *Mesocyclops leuckarti*, *Sinocalanus tenellus*, *Sinocalanus dorrii*, *Copepods nauplius*, but positively correlated with many Cyanophyta species (*Chroococcus minutus*, *Dactylococcopsis acicularis*, *Microcystis incerta*, *Merismopedia tenuissima*, *Merismopedia sinica* and *Lyngbya limnetica*). Based on our results, phytoplankton evenness was a better predictor of copepods abundance in meso-eutrophic lakes. These results provide new insights into the relationship between diversity and ecosystem functioning in aquatic ecosystems.

## 1. Introduction

The Earth ecosystem is experiencing an unprecedented rate of biodiversity loss as a result of global climate change, eutrophication, and overexploitation of natural resources [1,2,3]. Lake ecosystems are relatively vulnerable and the loss of biodiversity may cause catastrophic consequences, such as algae blooms [4,5]. Phytoplankton, the most important primary producer in lakes, is particularly sensitive to variations in environmental factors [5,6,7]. Understanding the effects of phytoplankton diversity on ecosystem functioning is essential to developing appropriate conservation strategies in aquatic ecosystems [5,8].

Aquatic ecosystems are special because their primary organisms (i.e., phytoplankton and zooplankton) are characterized by short generation times and efficient trophic transfer [9]. Phytoplankton diversity not only impacts the productivity, stability, resource use efficiency (measured as the amount of phytoplankton biomass produced per unit of phosphorus), and community turnover in its own trophic level [8,10,11,12], but also influences zooplankton through predator-prey interaction [13,14]. Ecologists have found that phytoplankton species richness could promote zooplankton abundance, a process known as “trophic overyielding” [14]. Filstrup et al. [12] showed that higher phytoplankton diversity (evenness) results in increased zooplankton resource use efficiency (measured as the amount of zooplankton biomass produced per unit of phytoplankton). It is commonly believed that communities with greater numbers of coexisting species are more resistant to predation [13]. The increase of producer species richness reduces the predation pressure and leads to smaller predator communities [15,16]. In addition, the relationship between biodiversity and ecosystem functioning varies with different metrics of diversity. For example, Ptacnik et al. [11] and Filstrup et al. [12] found contrary effects of phytoplankton species richness and evenness on community turnover rates. Therefore, the relationship between producer diversity and predator community is complex and has not reached a consistent conclusion, especially in natural aquatic ecosystems [8,13,14].

Predator-prey interaction between phytoplankton and zooplankton is an important mechanism in aquatic ecosystems [17,18]. The strength of the interaction is related to the trophic state of the lake: in oligotrophic lakes, zooplankton is mainly composed of small-sized individuals [19,20]; in mesotrophic lakes, zooplankton is dominated by efficient grazer copepods [19,20]; and in eutrophic lakes, phytoplankton mostly consists of Cyanophyta, which is predation-resistant and not efficiently transferred to zooplankton community [19,20]. While both phytoplankton diversity and biomass influence the predator community, debate remains as to which is a better predictor of zooplankton.

The copepod community is relatively larger in body size and is much more efficient in predation as compared with other zooplankton communities [19,20,21,22,23]. Furthermore, phytoplankton and copepods were believed to exert a strong influence on each other, especially in mesotrophic lakes [24,25]. In this study, the relationship between phytoplankton (evenness, species richness and biomass) and the abundance of copepods was analyzed using data from Lake Nansihu, which is a typical lake in North China containing a significant abundance of both phytoplankton and copepods. The purpose of this work was to explore the effects of phytoplankton diversity on the abundance of copepods in aquatic ecosystems.

## 2. Materials and Methods

### 2.1. Study Area

**Figure 1:**
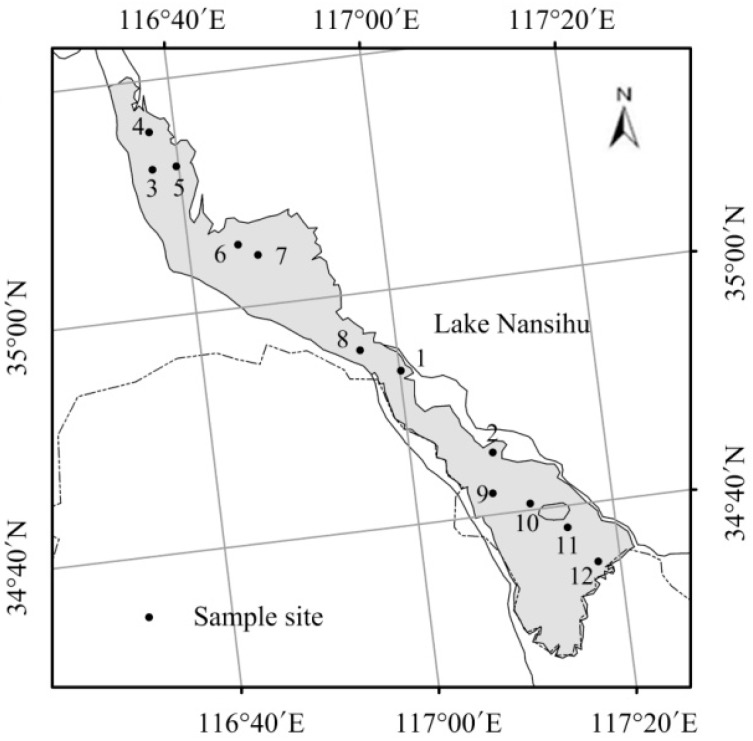
Location of Lake Nansihu and sampling sites in the lake. The figure was made by ArcGIS version 10.0 (ESRI, Redlands, CA, USA).

Lake Nansihu (116°34′E–117°21′E, 34°27′N–35°20′N), located in the northern of the Huai River Basin, is the largest freshwater lake in North China (Figure 1). The lake actually comprises four connected lakes: Nanyang, Dushan, Zhaoyang and Weishan. The total water area is 1266 km^2^ and the total capacity is 6.37 × 10^9^ m^3^. The average water depth of the lake is 1.5 m and it is categorized as a shallow, open and plain grassland lake. The lake experiences a warm temperate monsoon climate with an annual average temperature of 13.7 °C. Annual rainfall varies from 550 mm to 720 mm, with nearly 60% of precipitation occurring during the summer rainy season.

In the early 1980s, the lake was in a healthy state and contained 116 phytoplankton genera and 249 zooplankton species [26,27,28]. Since the late 1980s, pollution from external untreated industrial wastewater and agricultural runoff increased [27]. In 2000, the mean concentrations of total nitrogen (TN) and total phosphorus (TP) were 3.7 mg/L and 0.15 mg/L, respectively [26]. Plankton diversity decreased to the lowest level with only 36 phytoplankton species and 28 zooplankton species in 2002 [28]. Lake Nansihu was selected as a water delivery channel and storage lake for the great South-to-North Water Diversion Project in 2002. Then many ecological restoration projects were implemented to improve water quality. Mean concentrations of TN and TP had decreased to 1.01 mg/L and 0.09 mg/L in 2010 [29]. A total of 86 phytoplankton species and 52 zooplankton species were identified in 2007, which was much larger than that in 2002 [28]. There were six copepod species in the lake and their mean abundance was 70 ind./L in 2010 [30]. Lake Nansihu is currently in a meso-eutrophic state and the main health risk is algae blooms [31].

### 2.2. Sampling and Measurements

Data were collected from 12 sampling sites evenly distributed throughout the lake (Figure 1). Field investigations were conducted in early April, July, September, and late November from 2011 to 2014. Measurements and samplings were carried out between 8:00 A.M. and 10:00 A.M. along the same route.

For phytoplankton samples, equivalent amount of water was collected at depth of 0.5 m and 1.0 m using a water sampler, and then 1 L of the well-mixed water was taken. The samples were preserved with acidified Lugol’s solution for 24 h and condensed to 30 mL. Finally, a 0.1 mL-aliquot of the condensed sample was added to a phytoplankton counting box to allow for the identification and quantification of species [32]. The biomass of each phytoplankton species was calculated by cell volume [33]. Phytoplankton species richness was calculated as the number of species identified for each sample site.

For copepod samples, an equivalent amount of water was collected at depth of 0.5 m and 1.0 m using a water sampler, and then 1 L of the well-mixed water was taken. The samples were preserved with formaldehyde (4%) for 24 h. Then the samples were condensed to 50 mL and 1 mL of the condensed sample was used to identify and count the individuals of each copepod species under a microscope. Copepod abundance was expressed as the number of individuals per liter (ind./L).

Environmental factors were investigated at the same time with plankton in the four seasons of 2013. Water temperature (WT), dissolved oxygen (DO), and pH were measured in situ using YSI Professional Plus (YSI Incorporated, Yellow Springs, OH, USA) at each of the 12 sites. Water transparency (SD) was measured with a Secchi disk. Water quality samples were collected using a Tygon tube water sampler at 0.30 m under the water surface. Samples were stored in acid-cleaned glass bottles at 4 °C and filtered through a 0.45-μm acetate filter for subsequent analysis. The concentration of TN was measured via potassium persulfate oxidation-UV spectrophotometry, and TP was determined by Mo-Sb Anti-spectrophotometry method.

### 2.3. Calculation of Evenness and Statistical Analysis

Phytoplankton evenness was expressed using Pielou’s Evenness Index [34]:

(1) $$H=-\sum_{i=1}^{S}P_{i}\ln P_{i}$$

(2) $$P_{i}=N_{i}/N$$

(3) J=H/lnS

where *P_i_* is the biomass proportion of phytoplankton species *i* in the sample, *S* is the species richness, and *J* is Pielou’s Evenness Index.

The differences of environmental factors in different seasons and sites were analyzed via one-way ANOVA. Prior to analysis, the Kolmogorov-Smirnov method was used to test whether data were normally distributed, and the Bartlett test was performed to assess the homogeneity of variance. Post-hoc comparisons were applied using the Turkey HSD test at a significance level of 0.05 (function “glht” within the “multcomp” package). The correlation coefficients among copepods abundance, phytoplankton species richness, evenness and biomass were calculated by linear Pearson correlation.

Considering that the 12 sites had different environmental factors and phytoplankton conditions, we separately analyzed the relationships between phytoplankton (evenness, species richness and biomass) and copepods abundance using a Linear Mixed Effects Model with a maximum-likelihood estimator (function “lme” with “method = ML” within the “nlme” package in *R* 3.2.3). The 12 sites were included in the model as a random factor to correct for differences between sites.

Principal components analysis (PCA) was used to test the similarity of plankton community composition at different sites and whether there were apparent spatial characteristics. Mean values of phytoplankton and copepods species abundance at each site during the research time were used as the species matrix. The relationships among plankton species composition and environmental variables were analyzed using canonical correspondence analysis (CCA). The environmental matrix for CCA was including the values of WT, DO, pH, SD, TN, TP, phytoplankton biomass, species richness and evenness in the four seasons of 2013. Species matrix for CCA used the plankton data in the four seasons of 2013. Plankton species were selected for CCA through the following standards: the species appeared in at least 30% of the samples and contributed to ≥1% of total plankton abundance in at least one sample. All the variables were transformed by log_10_(x+1) except for pH. Both PCA and CCA were conducted using Canoco for Windows 4.5 (Biometris, Wageningen, The Netherlands), and the figures were drawn using Canodraw for Windows (Biometris, Wageningen, The Netherlands).

## 3. Results

### 3.1. Variations of the Environmental Factors

Mean WT at the 12 sites ranged from 21.4 °C to 22.2 °C and there were no significant differences among these sites (*F_(11, 36)_* = 0.04, *p* = 0.95). Mean WT varied seasonally (*F_(3, 44)_* = 441.9, *p* < 0.001) and had a significantly higher value in summer than in spring (*p* < 0.001), autumn (*p* < 0.001), and winter (*p* < 0.001). The DO concentration in the lake was relatively high and there were significant differences among the 12 sites (*F_(11, 36)_* = 1.85, *p* < 0.05), with mean value at Site 9 significantly lower than that at Site 3 (*p* = 0.007) and Site 7 (*p* = 0.022). Mean DO concentrations in the lake were similar during the spring, summer, and autumn (*F_(2, 33)_* = 0.530, *p* = 0.594). However, in winter the mean concentration of DO was significantly higher than that in autumn (*p* < 0.05). Most of the sites in the lake showed weak alkaline with a variation of pH from 7.48 to 7.94 (Table 1). Values of pH showed significant seasonal variation (*F_(3, 44)_* = 6.67, *p* = 0.001), with summer pH significantly higher than that in spring (*p* = 0.032) and winter (*p* < 0.001). The lake had a low SD, with mean values lower than 1 m at all 12 sites (Table 1), and there were no significant seasonal variations in mean transparent values (*F_(3, 44)_* = 1.96, *p* = 0.135). The nutrient concentrations in the lake were enriched, as shown in Table 1. The mean concentration of TN ranged between 0.72 mg/L and 1.59 mg/L at different sites, and there were significant differences among the 12 sites for TN (*F_(11, 36)_* = 2.37, *p* < 0.05). The TN concentration at Site 2 was significantly higher than that at Site 3 (*p* = 0.021) and Site 6 (*p* = 0.007). Besides, the TN concentration in the summer was significantly higher than that in spring (*p* = 0.024). There were no significant differences among all sites for TP (*F_(11, 36)_* = 0.64, *p* = 0.28). However, there were significant seasonal variations among TP concentrations (*F_(3, 44)_* = 15.5, *p* < 0.001), with the mean TP concentration in summer significantly higher than that in the winter (*p* < 0.001).

### 3.2. Seasonal and Spatial Variations of Phytoplankton Community

In total, 138 phytoplankton species belonging to 78 genera and eight phyla were identified between 2011 and 2014. The phytoplankton community included 60 Chlorophyta species, 33 Bacillariophyta species, 20 Cyanophyta species, and 14 Euglenophyta species. Species belonging to other communities were relatively rare.

**Figure 2:**
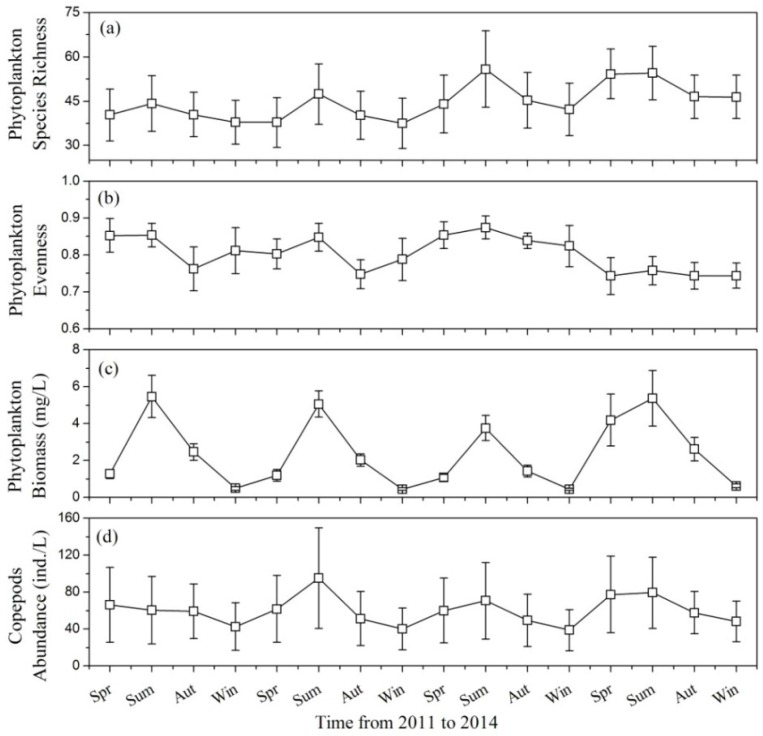
Seasonal variations in (**a**) phytoplankton species richness; (**b**) phytoplankton evenness; (**c**) phytoplankton biomass; and (**d**) copepods abundance. Values are expressed as mean ± standard deviation of the 12 sample sites. Spr: spring, Sum: summer, Aut: autumn, Win: winter.

The mean values of phytoplankton species richness varied seasonally from 37.5 (winter 2012) to 55.8 (summer 2013), with annual maximum values in summer and minimum values in winter (Figure 2a). In different sample sites, phytoplankton species richness ranged between 22 and 88 with large seasonal standard deviations (varied between 7.29 and 12.96; Figure 2a). Phytoplankton evenness was high with mean values ranging between 0.74 (spring 2014) and 0.87 (summer 2013; Figure 2b). Variation in phytoplankton evenness was irregular and sometimes significant between adjacent seasons (e.g., in 2012 mean phytoplankton evenness ranged from 0.85 in summer to 0.75 in autumn; Figure 2b).

**Figure 3:**
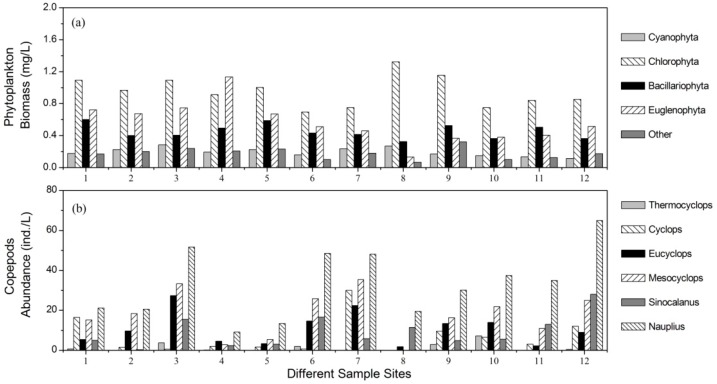
The community compositions of (**a**) phytoplankton and (**b**) copepods at different sites.

Mean phytoplankton biomass across the study was 2.19 mg/L; however, there were significant seasonal variations (Figure 2c), with mean phytoplankton biomass varying between 0.44 mg/L (winter 2013) and 5.46 mg/L (summer 2011). Chlorophyta was the dominant community in the lake with a mean biomass of 0.94 mg/L, which accounted for 43.0% of the total phytoplankton biomass. The mean biomasses of Bacillariophyta and Euglenophyta were almost equal (0.45 mg/L and 0.43 mg/L respectively). Cyanophyta had a relatively lower mean biomass (0.20 mg/L) and its proportion was 8.92%. Phytoplankton biomasses were notably lower at Sites 6 and 10 than they were at other sites (Figure 3a). Site 8 had the highest proportion of Cyanophyta biomass, and its value reached 12.7%. The biomasses of Chlorophyta at Sites 8 and 9 were higher than that at other sites (Figure 3a). Site 4 had the highest value of Euglenophyta biomass.

### 3.3. Seasonal and Spatial Variations in Copepods Abundance

In total, 12 copepod species were identified, including *Thermocyclops hyalinus*, *Thermocyclops taihokuensis*, *Thermocyclops kawamurai*, *Cyclops vicinus, Tropocyclops prasinus jerseyensis, Eucyclops serrulatus, Mesocyclops leuckarti, Eucyclops speratus, Sinocalanus tenellus, Sinocalanus dorrii, Cletocamptus*, and *Copepods nauplius*. The dominant species were *C. vicinus, M. leuckarti, S. dorrii*, and *Copepods nauplius*.

Mean copepod abundance ranged from 38.6 ind./L (winter 2013) to 95.1 ind./L (summer 2012) in different seasons (Figure 2d), and was relatively higher in spring and summer than in autumn and winter (Figure 2d). Copepod abundance also varied significantly between different sample sites, as shown by the large standard deviation values (Figure 2d). Mean copepod abundance at Sites 3, 7 and 12 were much higher than those at the other sites (Figure 3b). The mean biomasses of *Cyclops* and *Mesocyclops* reached their maximum values at Site 7 (Figure 3b); however, the maximum values for *Eucyclops* and *Sinocalanus* were observed at Sites 3 and 12, respectively.

**Figure 4:**
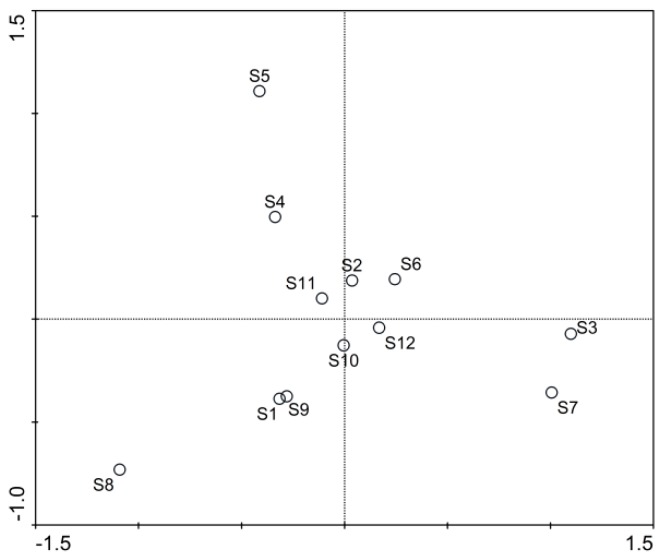
PCA biplot of spatial distribution characters for plankton community in Lake Nansihu. S1 to S12 were standing for the 12 sample sites in the lake.

The results of the PCA for the first two components are presented in the biplot (Figure 4). The eigenvalues of the first two axes were 0.395 and 0.275, respectively, and the contribution of the first two principal components to the percentage of variance was 67.1%. The first principal component was primarily driven by both phytoplankton and copepods species, while the second principal component was primarily driven by *Cyclotella*, *Synedra*, *Lyngbya*, *Closterium*, *Scenedesmus* genera and some other Chllorophyta species. Sites 3 and 7 were similar in plankton composition and were distributed in the positive direction of the first principal component (Figure 4). Sites 4 and 5 were distributed within the second quadrant, and both had low copepods abundance and similar phytoplankton compositions (Figure 3). Site 8 was located within the third quadrant and the remaining sites were mainly distributed near the origin of the coordinates (Figure 4). Thus, there were apparent differences for plankton community composition among the 12 sites. However, plankton community composition did not show apparent latitudinal distribution.

### 3.4. Relationship between Phytoplankton Evenness and Copepods Abundance

**Figure 5:**
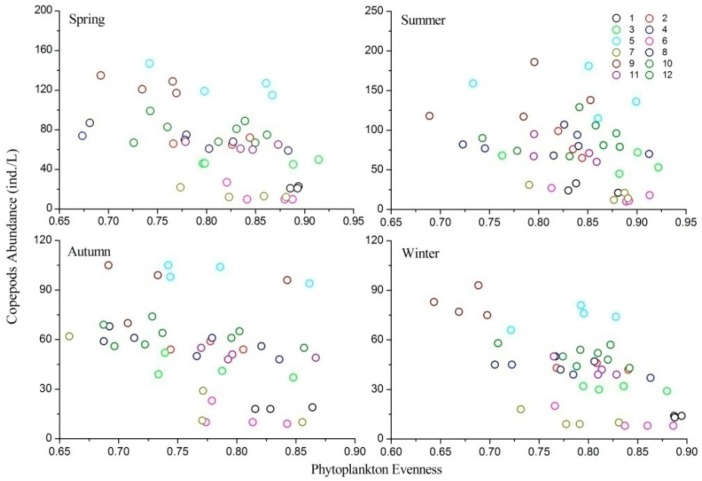
Relationships between phytoplankton evenness and copepod abundance in different seasons.

Phytoplankton evenness was negatively correlated with copepod abundance in all four seasons (spring, *R* = −0.532, *p* < 0.001; summer, *R* = −0.355, *p* < 0.05; autumn, *R* = −0.372, *p* < 0.05; winter, *R* = −0.616, *p* < 0.001). A Linear Mixed Effects Model showed that phytoplankton evenness was significantly correlated with the abundance of copepods throughout the year (Table 2; Figure 5), with regression slopes of −76.57 (*t* = −3.78, *p* < 0.001), −46.83 (*t* = −2.53, *p* = 0.021), −83.03 (*t* = −2.97, *p* = 0.005), and −44.16 (*t* = −2.39, *p* = 0.023), respectively.

### 3.5. Relationship between Phytoplankton Species Richness and Copepod Abundance

**Figure 6:**
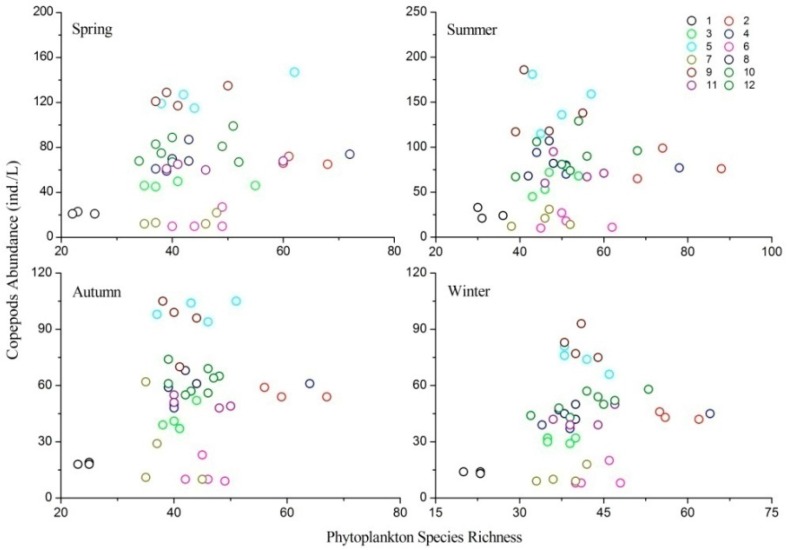
Relationships between phytoplankton species richness and copepod abundance in different seasons.

Linear Pearson correlation coefficients between phytoplankton species richness and copepod abundance in the four seasons were *R* = 0.236, *R* = 0.114, *R* = 0.213 and *R* = 0.262, respectively, which were all found to be insignificant (*p* > 0.05). The Linear Mixed Effects Model also showed that phytoplankton species richness had no significant correlation with the abundance of copepods (Table 3; Figure 6). The regression slopes in the four seasons were 0.468 (*t* = 1.410, *p* = 0.172), 0.027 (*t* = 0.066, *p* = 0.947), 0.002 (*t* = 0.001, *p* = 0.990) and 0.205 (*t* = 1.414, *p* = 0.167), respectively. All these regressions slopes were positive but not significant (*p* > 0.05).

### 3.6. Relationship between Phytoplankton Biomass and Copepods Abundance

**Figure 7:**
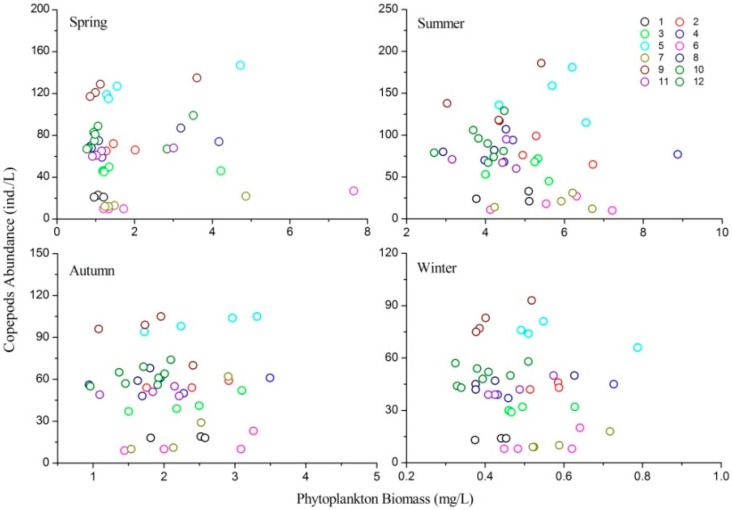
Relationships between phytoplankton biomass and copepod abundance in different seasons.

Pearson correlation coefficients between phytoplankton biomass and copepod abundance for the four seasons were *R* = 0.030, *R* = −0.146, *R* = −0.027, and *R* = −0.128, respectively, which were all insignificant (*p* > 0.05). However, the Linear Mixed Effects Model showed that the relationship between phytoplankton biomass and copepod abundance varied seasonally (Table 4; Figure 7). In spring, phytoplankton biomass had a positive correlation with copepod abundance and the regression slope was 3.597 (*t* = 6.715, *p* < 0.001). However, the regression slopes in summer, autumn, and winter were all positive, none were significant (Table 4). The correlation coefficients between phytoplankton evenness and biomass for the four seasons (spring, summer, autumn, and winter) were *R* = −0.153, *R* = −0.103, *R* = −0.211, and *R* = −0.171, respectively. In addition, the correlation coefficients between phytoplankton species richness and biomass were always insignificant (spring: *R* = 0.213; summer: *R* = 0.106; autumn: *R* = 0.057; winter, *R* = 0.206). These results showed that phytoplankton evenness and species richness had no significant correlation with biomass.

### 3.7. Influence of Environmental Factors and Phytoplankton on Copepods Abundance

Results of CCA showed that the first two environmental factors axes were all vertical, and the first two species axes were nearly perpendicular. The eigenvalues of the first two axes were 0.116 and 0.042, respectively. These axes explained 62.1% of the total variance of the plankton species listed in Table 5. The correlation coefficients between the first two environmental axes and species axis were 0.903 and 0.790. Species axis 1 was positively correlated with phytoplankton biomass (*R* = 0.703), but had a weak negative relationship with species richness (*R* = −0.210). Species axis 2 was negatively correlated with phytoplankton evenness (*R* = −0.533), SD (*R* = −0.476) and WT (*R* = −0.406), but had a weak positive relationship with DO (*R* = 0.173).

**Figure 8:**
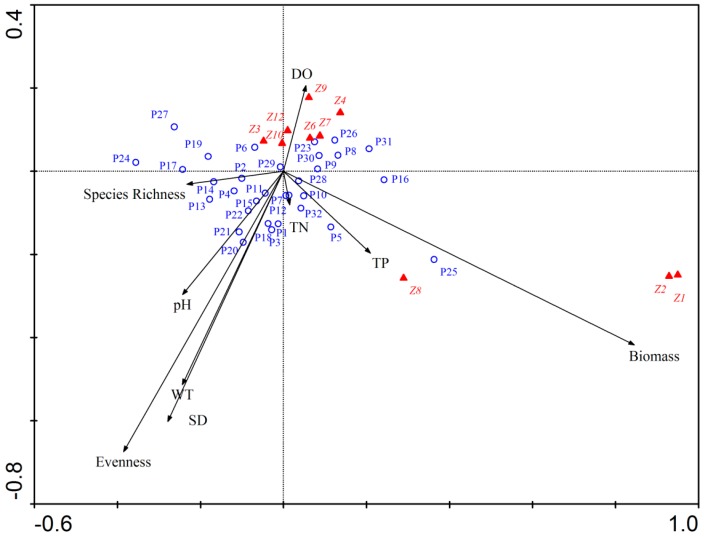
CCA biplot for the relationships among plankton species composition and environmental variables.

Copepod species *T. kawamurai* (*Z3*), *C. vicinus* (*Z4*), *E. Serrulatus* (*Z6*), *M. leuckarti* (*Z7*), *S. tenellus* (*Z9*), *S. dorrii* (*Z10*), *Copepods nauplius* (*Z12*) were distributed in the positive direction of axis 2, and their abundance was negatively correlated with phytoplankton evenness. However, *T. hyalinus* (*Z1*), *T. taihokuensis* (*Z2*) and *E. speratus* (*Z8*) were distributed in the fourth quadrant, and their abundance was primarily influenced by phytoplankton biomass (Figure 8). The biomasses of Cyanophyta species *C. minutus* (*P15*), *M. incerta* (*P18*), *M. tenuissima* (*P20*), *M. sinica* (*P21*), and *L. limnetica* (*P22*) had a weak positive relationship with phytoplankton evenness. In addition, phytoplankton evenness had positive relationships with the biomasses of Euglenophyta species *D. acutum* (*P1*), *P. helicoides* (*P3*), Bacillariophyta species *F. capucina* (*P11*). Chlorophyta species *C. gracile* (*P26*), *S. dimorphus* (*P30*) and *C. tetrapedia* (*P31*) had a negative relationship with phytoplankton evenness. Phytoplankton species richness was positively correlated with Euglenophyta species *E. lucens* (*P2*) and *E. mutabilis* (*P4*), Cyanophyta species *O. princeps* (*P13*) and *O. tenuis* (*P14*).

Concentration of DO had positive influence on most copepods species but had a negative relationship with many phytoplankton species as shown in Figure 8. Nutrient concentration in the lake had relatively weak influence on plankton community (Figure 8). However, TP had a strong correlation with the abundance of *E. speratus* (*Z8*) and phytoplankton biomass. SD, WT and pH were the main environmental factors that influence plankton community and they all positively correlated with phytoplankton evenness (Figure 8). The values of pH was strongly correlated with Cyanophyta species *C. minutus* (*P15*), *M. tenuissima* (*P20*), *M. sinica* (*P21*), and *L. limnetica* (*P22*). WT and SD had a strong positive relationship with Cyanophyta species *M. incerta* (*P18*), *M. tenuissima* (*P20*) and Euglenophyta species *D. acutum* (*P1*) and *P. helicoides* (*P3*).

## 4. Discussion

Phytoplankton diversity is thought to influence zooplankton through predator-prey interaction [13,14]. Based on a manipulated experiment, Striebel et al. [14] showed that phytoplankton communities with larger species richness could generate higher *Daphnia* growth rates and abundance under constant phytoplankton biomass. However, the results of this study found no significant relationship between phytoplankton species richness and copepod abundance in the natural aquatic ecosystem of Lake Nansihu (Figure 6; Table 3). Both phytoplankton species richness and evenness were insignificantly correlated with biomass. Phytoplankton species richness ranged from 1 to 10 in the experiment of Striebel et al. [14], while in this study, it varied between 22 and 88. This discrepancy suggests that the “trophic overyielding” is only observed in the simple man-made community, and is not appeared in complex and highly evolutionary ecosystems. Therefore, the relationship between phytoplankton species richness and zooplankton abundance has not formed a consistent conclusion and further investigations are needed.

Filstrup et al. [12] found that higher phytoplankton evenness resulted in larger zooplankton resource use efficiency. However, the results of this study showed a negative relationship between phytoplankton evenness and copepod abundance. In the research of Filstrup et al. [12], plankton samples were collected from heavy eutrophic lakes and phytoplankton evenness ranged from 0 to 0.75. Phytoplankton community with low evenness was dominated by few Cyanophyta genera and the biomass proportion of Cyanophyta was higher than 75% at most sites in their study [12]. Cyanophyta was predation-resistant and not efficiently transferred to zooplankton community [12,19,20]. Thus, phytoplankton evenness was positively related to zooplankton resource use efficiency [12]. Lake Nansihu was meso-eutrophic and phytoplankton evenness varied between 0.64 and 0.93. The mean biomass of Cyanophyta was 0.20 mg/L and its proportion was 8.92%, which were relatively lower than other phyla. WT, SD and pH were the main environmental factors that influence plankton community in the lake while the effects of nutrients were relatively weak (Figure 8). WT, SD and pH had a strong positive relationship with many Cyanophyta species (Figure 8). The increase of Cyanophyta biomass increased the uniformity of the phytoplankton community. As a result, there was a positive relationship between phytoplankton evenness and the biomass of many Cyanophyta species (Figure 8). Thus phytoplankton community with higher level of evenness comprised larger biomass of Cyanophyta species, which were more resistant to predation by copepods and lead to a negative relationship between phytoplankton evenness and copepods abundance. These results suggest that the relationship between biodiversity and ecosystem functioning is related to the trophic state of a lake. In addition, copepod abundance is likely to reach a high level at two conditions: intermediate level of evenness and low biomass proportion of Cyanophyta.

When analyzing the relationship between biodiversity and ecosystem functioning, ecologists found that the influence of plant species richness and evenness may be different, especially in aquatic ecosystems [11,12,35]. Ptacnik et al. [11] obtained a positive correlation between phytoplankton species richness and resource use efficiency, while Filstrup et al. [12] found that phytoplankton evenness had a negative influence on resource use efficiency. In this study, we discovered that phytoplankton evenness had a negative correlation with copepod abundance while no effect of species richness was apparent. Evenness generally responds more rapidly to the fluctuation of environmental factors than species richness because many species become rare before becoming locally extinct [35,36]. Rare species may have little impact on ecosystem functioning, and therefore species richness would often overestimate the importance of rare species [12]. The results in this study showed that copepod abundance was more sensitive to the variation of phytoplankton evenness than species richness, which was consistent with previous studies [12,35,36]. On the basis of these results, a distinction should be made between species richness and evenness when analyzing the relationship between phytoplankton diversity and ecosystem functioning.

It is believed that the predator-prey interaction between phytoplankton and zooplankton is a determinable mechanism in aquatic ecosystems [17,18]. The increase of phytoplankton biomass will promote the growth of zooplankton, and in turn leading to greater phytoplankton consumption [37,38]. The relationships between the two variables are related to the trophic condition of a lake: in oligotrophic lakes, zooplankton is dominated by small-sized individuals and phytoplankton biomass will benefit the growth of zooplankton; in eutrophic lakes, phytoplankton is generally dominated by cyanobacteria, which is grazing-resistant species and phytoplankton biomass will limiting the growth of zooplankton; in mesotrophic lakes, zooplankton is often dominated by efficient grazer *Daphnia* and copepods, the relationship between phytoplankton and zooplankton biomasses is complex [19,20]. In this study, most of the sites in Lake Nansihu are in mesotrophic or light-eutrophic state (Table 1), and we found no apparent relationship between phytoplankton biomass and copepods. Long-term observations of predator-prey systems have sometimes showed complex or even chaotic plankton abundance dynamics [37,38]. Therefore, a coupled oscillation between the biomasses of phytoplankton and zooplankton may be a good relationship.

In lake ecosystems, zooplankton is generally influenced by both bottom-up and top-down effects [39]. Fish species have great influence on plankton and are usually used to control algae blooms through trophic cascading [39]. In Lake Nansihu, *Carassius auratus* was the main fish species, with a biomass proportion of 69.15% [40], and the biomass proportion of Cyprinidae was higher than 90% of total fish biomass. Specziár et al. [41] observed that copepod biomass accounted for 4.4% of the total gut content of Cyprinidae. Thus in Lake Nansihu, copepods were mainly influenced by bottom-up effects and the effect of fish species was relatively weak.

Both biodiversity and ecosystem functioning are multidimensional and with a wide variety of definitions, therefore, the relationship between them is a complex and long-term topic [3,42]. However, until now, little has been known about how phytoplankton diversity affects ecosystem functioning. Given the importance of aquatic ecosystems and the special ecophysiological traits of plankton community [8,43,44], further investigations in this field will be critical for improving our understanding of these phenomena.

## 5. Conclusions

In this study, the relationship between phytoplankton (evenness, species richness and biomass) and copepod abundance was analyzed at Lake Nansihu, China. The main findings of the present research can be summarized in the following conclusions:

(1) A total of 138 phytoplankton species belonging to 78 genera and eight phyla were identified, including 60 Chlorophyta species, 33 Bacillariophyta species, and 20 Cyanophyta species, phytoplankton biomass varied from 0.44 mg/L to 5.46 mg/L and Chlorophyta was the dominant community;
(2) There were 12 copepod species in the lake and their mean abundance ranged between 38.6 ind./L and 95.1 ind./L in different seasons;
(3) Copepod abundance was significantly decreased with increasing phytoplankton evenness throughout the year, and both phytoplankton species richness and biomass had no significant correlation with the abundance of copepods;
(4) The influence of phytoplankton species richness and evenness was different and most copepods species were more sensitive to the variation of phytoplankton evenness than species richness.

The results of this study provide important new insights into the relationship between phytoplankton diversity and ecosystem functioning.

## Figures and Tables

**Table 1:** Environmental factors for each sample site, values are expressed as mean ± standard deviation. WT: water temperature; DO: dissolved oxygen; SD: Water transparency; TN: total nitrogen; TP: total phosphorus.

Sites	WT (°C)	DO (mg/L)	pH	SD (cm)	TN (mg/L)	TP (mg/L)
1	22.14 ± 10.03	10.47 ± 2.94	7.83 ± 0.69	40.75 ± 9.22	1.19 ± 0.31	0.24 ± 0.11
2	21.94 ± 10.03	8.22 ± 1.90	7.69 ± 0.25	58.00 ± 5.89	1.22 ± 0.21	0.09 ± 0.04
3	21.38 ± 9.96	9.27 ± 0.36	7.55 ± 0.66	40.25 ± 7.68	0.80 ± 0.18	0.12 ± 0.09
4	22.15 ± 9.58	9.13 ± 1.23	7.91 ± 0.46	87.88 ± 10.82	1.17 ± 0.15	0.11 ± 0.06
5	21.59 ± 9.27	8.43 ± 1.19	7.88 ± 0.51	65.38 ± 9.33	1.18 ± 0.24	0.10 ± 0.06
6	21.93 ± 10.24	9.49 ± 1.34	7.69 ± 0.75	89.87 ± 11.91	0.72 ± 0.15	0.11 ± 0.09
7	21.61 ± 11.01	9.59 ± 1.41	7.88 ± 0.62	49.00 ± 8.25	1.03 ± 0.25	0.17 ± 0.10
8	21.61 ± 9.48	7.31 ± 1.05	7.70 ± 0.37	48.02 ± 10.31	1.59 ± 0.34	0.24 ± 0.11
9	22.73 ± 6.50	6.74 ± 1.21	7.51 ± 0.36	72.63 ± 12.42	1.30 ± 0.28	0.13 ± 0.06
10	21.16 ± 9.95	7.63 ± 1.83	7.48 ± 0.34	38.00 ± 8.36	1.27 ± 0.37	0.14 ± 0.10
11	21.21 ± 9.72	9.11 ± 1.89	7.61 ± 0.47	46.62 ± 4.23	1.36 ± 0.24	0.11 ± 0.09
12	21.47 ± 9.79	11.01 ± 1.69	7.94 ± 0.53	74.87 ± 9.55	1.16 ± 0.31	0.18 ± 0.11

**Table 2:** Linear Mixed Effects Model analyses for the relationship between phytoplankton evenness and copepod abundance in different seasons.

Seasons	Variables	Value	Standard Error	Degree of Freedom	*t*-Value	*p*-Value
Spring	Intercept	127.3	19.43	35	6.55	<0.001
	Slope	−76.57	20.28	35	−3.78	<0.001
Summer	Intercept	114.82	49.67	35	2.31	0.027
	Slope	−46.83	18.50	35	−2.53	0.021
Autumn	Intercept	117.83	22.86	35	5.15	<0.001
	Slope	−83.03	27.92	35	−2.97	0.005
Winter	Intercept	76.58	15.90	35	4.82	<0.001
	Slope	−44.16	18.45	35	−2.39	0.023

**Table 3:** Linear Mixed Effects Model analyses for the relationship between phytoplankton species richness and copepod abundance in different seasons.

Seasons	Variables	Value	Standard Error	Degree of Freedom	*t*-Value	*p*-Value
Spring	Intercept	44.34	12.05	35	3.681	<0.001
	Slope	0.468	0.332	35	1.410	0.172
Summer	Intercept	74.32	23.68	35	3.138	0.003
	Slope	0.027	0.400	35	0.066	0.947
Autumn	Intercept	53.35	15.67	35	3.404	0.002
	Slope	0.002	0.317	35	0.001	0.990
Winter	Intercept	33.07	8.739	35	3.784	<0.001
	Slope	0.205	0.145	35	1.414	0.167

**Table 4:** Linear Mixed Effects Model analyses for the relationship between phytoplankton biomass and copepod abundance in different seasons.

Seasons	Variables	Value	Standard Error	Degree of Freedom	*t*-Value	*p*-Value
Spring	Intercept	58.28	11.00	35	5.298	<0.001
	Slope	3.597	0.536	35	6.715	<0.001
Summer	Intercept	71.05	19.01	35	3.738	<0.001
	Slope	0.941	2.969	35	0.317	0.753
Autumn	Intercept	39.52	9.504	35	4.158	<0.001
	Slope	6.564	3.551	35	1.848	0.085
Winter	Intercept	30.99	7.684	35	4.033	<0.001
	Slope	11.29	7.714	35	1.463	0.161

**Table 5:** Codes and Latin names of plankton species for CCA.

Code	Latin Name	Code	Latin Name	Code	Latin Name
P1	*Distigma acutum*	P2	*Euglena lucens*	P3	*Phacus helicoides*
P4	*Euglena mutabilis*	P5	*Euglena caudata*	P6	*Cyclotella* sp.
P7	*Melosira granulata*	P8	*Nitzschia sublinearis*	P9	*Synedra* sp.
P10	*Navicula simplex*	P11	*Fragilaria capucina*	P12	*Monallantus brevicylindrus*
P13	*Oscillatoria princeps*	P14	*Oscillatoria tenuis*	P15	*Chroococcus minutus*
P16	*Chroococcus tenax*	P17	*Dactylococcopsis acicularis*	P18	*Microcystis incerta*
P19	*Phormidium tenue*	P20	*Merismopedia tenuissima*	P21	*Merismopedia sinica*
P22	*Lyngbya limnetica*	P23	*Lyngbya major*	P24	*Lyngbya contorta*
P25	*Cylindrospermum stagnale*	P26	*Closterium gracile*	P27	*Chlorella* sp.
P28	*Schroederia setigera*	P29	*Scenedesmus quadricauda*	P30	*Scenedesmus dimorphus*
P31	*Crucigenia tetrapedia*	P32	*Actinastrum* sp.	Z1	*Thermocyclops hyalinus*
Z2	*Thermocyclops taihokuensis*	Z3	*Thermocyclops kawamurai*	Z4	*Cyclops vicinus*
Z5	*Tropocyclops prasinus jerseyensis*	Z6	*Eucyclops serrulatus*	Z7	*Mesocyclops leuckarti*
Z8	*Eucyclops speratus*	Z9	*Sinocalanus tenellus*	Z10	*Sinocalanus dorrii*
Z11	*Cletocamptus*	Z12	*Copepods nauplius*		
